# Comparison of Geostatistical Kriging Algorithms for Intertidal Surface Sediment Facies Mapping with Grain Size Data

**DOI:** 10.1155/2014/145824

**Published:** 2014-02-13

**Authors:** No-Wook Park, Dong-Ho Jang

**Affiliations:** ^1^Department of Geoinformatic Engineering, Inha University, Incheon 402-751, Republic of Korea; ^2^Department of Geography, Kongju National University, Kongju 314-701, Republic of Korea

## Abstract

This paper compares the predictive performance of different geostatistical kriging algorithms for intertidal surface sediment facies mapping using grain size data. Indicator kriging, which maps facies types from conditional probabilities of predefined facies types, is first considered. In the second approach, grain size fractions are first predicted using cokriging and the facies types are then mapped. As grain size fractions are compositional data, their characteristics should be considered during spatial prediction. For efficient prediction of compositional data, additive log-ratio transformation is applied before cokriging analysis. The predictive performance of cokriging of the transformed variables is compared with that of cokriging of raw fractions in terms of both prediction errors of fractions and facies mapping accuracy. From a case study of the Baramarae tidal flat, Korea, the mapping method based on cokriging of log-ratio transformation of fractions outperformed the one based on cokriging of untransformed fractions in the prediction of fractions and produced the best facies mapping accuracy. Indicator kriging that could not account for the variation of fractions within each facies type showed the worst mapping accuracy. These case study results indicate that the proper processing of grain size fractions as compositional data is important for reliable facies mapping.

## 1. Introduction

As transition zones where the land and sea meet, tidal flats are highly productive zones with specific ecosystems. Especially, sediment properties in tidal flats, such as grain size and sediment facies, are related to sediment transport and stability and affect intertidal habitats [1, 2]. These properties can therefore be useful information sources for effective coastal zone management.

The characteristics of intertidal surface sediments are usually described using grain size data. To map sediment grain size distributions, surface sediment samples are first collected via field survey and the information on grain size is then generated through laboratory experiments. From the grain size data, the sediment fractions, as denoted by percentages of sand, silt, and clay, are generally obtained and sedimentary facies are then classified by applying a certain classification scheme, such as Shepard's rule [3]. Due to the short exposure time of intertidal surfaces and the limitations of sampling cost, however, only limited numbers of field samples are generally available. Thus, spatial prediction or interpolation is usually applied to generate exhaustive information over the study area. Many interpolation algorithms ranging from deterministic models (e.g., inverse distance weighting) to geostatistical kriging can be applied to this spatial prediction task. Especially, kriging, which can be regarded as a main part of geostatistics, has several advantages over the deterministic models. It can not only account for spatial patterns inherent to sample data such as spatial correlation and anisotropy, but also integrate various types of spatial data such as categorical and/or continuous data [4, 5].

Some kriging algorithms can be applied to intertidal surface sediment facies mapping using grain size data. The first possible approach is indicator kriging that can be applied directly to categorical data [6], because the target attribute for intertidal surface sediment facies mapping is categorical information. In this approach, sample data are first classified as sediment facies according to their grain size fractions and indicator transform is then applied. Spatial correlation information of indicator-coded binary data for each sediment class is used for indicator kriging.

As another approach, grain size fractions are first predicted at unsampled locations and the classification rule is finally applied to map sediment facies over the study area. Spatial correlation information is directly derived from each grain size component and used for the kriging algorithm. During spatial prediction of each grain component, however, special care should be taken due to the particular characteristics of the grain size fractions. The grain size fractions, which are usually expressed as the relative proportions, are regarded as compositional data that have both nonnegative values and a constant sum (e.g., 1 or 100%) [7]. Due to these constraints, spurious correlation is observed in compositional data, such that as one component decreases one or more of the other components should increase and vice versa. Although spatial auto- and cross-correlation information can be explained in cokriging, the direct application of conventional cokriging without consideration for these constraints may generate negative values and the sum of predicted component values may not be constant. For appropriate consideration of the characteristics of compositional data in spatial prediction, log-ratio based transformation is usually applied to the original compositional data prior to kriging analysis [8].

The spatial prediction of grain size fractions is not a final goal per se, but it should be regarded as a preliminary step toward intertidal sediment facies mapping. Log-ratio transformation is much more appropriate for compositional data analysis. However, prediction results are still subject to errors attached to the prediction and those errors may affect the subsequent facies mapping results, because prediction results are directly fed into the classification rule. Thus, the potential of the application of log-ratio transformation to spatial prediction of grain size fractions should be evaluated in terms of not only prediction accuracy of grain size fractions, but also facies mapping accuracy.

In relation to spatial prediction of compositional data and subsequent classification, several studies have been carried out for soil texture classes mapping [9, 10], seabed sediment texture classes mapping [11], and soil erodibility factor mapping [12]. These studies reported that if the special requirements of compositional data are not considered, spatial prediction of both fractions and classes will not generate reliable mapping results. To our knowledge, however, spatial prediction of sediment fractions as compositional data and the comparison with indicator kriging have not yet been conducted for intertidal surface sediment facies mapping.

The objective of this paper is to evaluate the predictive performance of the following three different kriging approaches for intertidal surface sediment facies mapping: (1) indicator kriging, (2) cokriging without data transformation, and (3) cokriging with additive log-ratio (alr) transformation. This performance evaluation is illustrated through a case study with a grain size data set acquired in the Baramarae tidal flat, Korea.

## 2. Study Area and Data

The application of different kriging algorithms for surface sediment facies mapping is compared via a case study in the Baramarae tidal flat, Korea. The study area is underlain by Precambrian metasediment, the Kyeonggi Metamorphic Complex, and several intrusive rocks [13]. In the study area, two sea stacks including the Halmi and Seomot isles, wave-cut platform and sand dunes have developed and blocked ocean waves, so large tidal flats have developed at an indentation in the coast. The west coastal areas face the open sea and are directly affected by ocean waves, so sand spits, beach, and coastal sand dunes have developed [1]. Meanwhile, much sediment has been continuously provided by the tidal currents in the eastern part of the coast, so large tidal flats were developed. The tides in the study area are semidiurnal, with a mean tidal range of 4.6 m. The mean tidal current velocities near the study area are 0.8 m/sec and 0.9 m/sec during flow tide and ebb tide, respectively [14].

**Figure 1:**
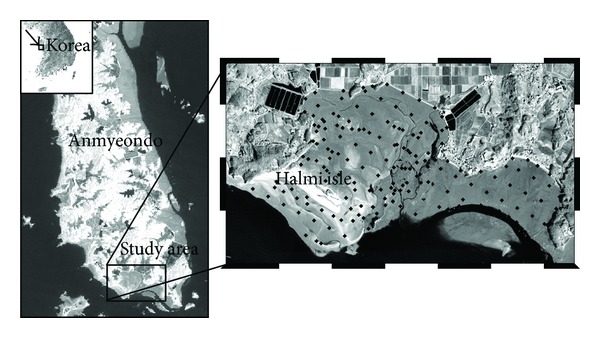
Location of the case study area. Black dots denote sampling sites.

In the study area, a field survey was conducted in February 2009, and 174 surface sediment samples were collected (Figure 1). A portable GPS (GARMIN 60CSx) was used for positioning of the sample locations. The sediment samples were sieved using a set of sieves and then analyzed using a Mastersizer 2000 at the laboratory to obtain sample fractions such as relative percentages of sand, silt, and clay. Detailed procedures for sample data processing at the laboratory can be found in Jang et al. [14].

## 3. Methods

Kriging, which is a generalized least-squares interpolation method, predicts attribute values at unsampled locations by using neighboring samples and spatial correlation information modeled by variogram [5, 15].

Suppose that there are *n* sediment samples with *D* components {*z*_*i*_(**u**_*α*_), *α* = 1,…, *n*, *i* = 1,…, *D*} in the study area. Each sample is a composition with *D* = 3 components, which are the percentages of sand, silt, and clay. In this case study, the following three different kriging algorithms are compared and evaluated: indicator kriging, cokriging without data transformation, and cokriging with alr transformation.

### 3.1. Indicator Kriging of Facies Types

The first kriging approach considered in this study is indicator kriging that attempts to model probabilities for all categories by considering neighboring indicator-coded values [6]. The indicator approach requires a preliminary coding of sample data into local prior probabilities. By directly applying the classification rule, sediment facies types are first identified at each sample location. According to the percentage of sand, each sample is classified into the following three facies types: sand flats (above 70%), mixed flats (30–70%), and mud flats (0–30%), after Folk [16]. Suppose that {*ω*_*k*_, *k* = 1,2, 3} is a set of the above three sediment facies types. Then the sediment facies type at sample locations (*ω*(**u**_*α*_)) is coded into three binary indicator probabilities (*I*(**u**_*α*_; *ω*_*k*_)) as follows:

(1) I(uα;ωk)={1if  ω(uα)=ωk,0otherwise,k=1,2,3.

The binary indicator variable is interpreted as the probability for a certain sediment facies type to prevail at a particular location: the probability is 1 if it prevails and 0 if it does not. After indicator coding, variogram modeling for each of the three indicator variables is conducted to incorporate spatial correlation information into the indicator kriging system.

The conditional probability *p*(**u**; *ω*_*k*_) at any location is regarded as the conditional expectation of the indicator random function *I*(**u**; *ω*_*k*_). In this paper, it is estimated by ordinary kriging using neighboring indicator data:

(2) p∗(u;ωk)=I∗(u;ωk)=∑α=1mλα(u;ωk)I(uα;ωk),with  ∑α=1mλα(u;ωk)=1 k=1,2,3,

where *m* is the number of neighboring samples within a predefined search neighborhood and *λ*_*α*_(**u**; *k*) is an ordinary kriging weight assigned to the neighboring indicator values at a prediction location **u**.

The ordinary kriging weight is calculated by solving the following ordinary kriging system [5]:

(3) ∑β=1mλβ(u;ωk)CI(uα−uβ;ωk)+μ(u;ωk)  =CI(uα−u;ωk) α=1,…,m,  k=1,2,3,∑α=1mλα(u;ωk)=1,

where *μ*(**u**; *k*) is the Lagrange parameter for meeting the constraint on the weights such that the sum of the kriging weights is 1. *C*_*I*_(**u**_*α*_ − **u**_*β*_; *ω*_*k*_) and *C*_*I*_(**u**_*α*_ − **u**; *ω*_*k*_) are the data-to-data covariance and the data-to-sample indicator covariance for each facies type *ω*_*k*_, respectively.

Kriging is a nonconvex interpolator and indicator kriging is independently and repeatedly applied to the set of *K* facies types. Thus, the estimated probability may be outside the interval [0,1] and the constant sum constraint may not be satisfied [17]. This order relation violation is corrected by resetting any probability outside the interval to the closest bound, 0 or 1, and standardization.

After estimating the conditional probability at unsampled locations, the final sediment facies types over the study area are mapped by applying the maximum a posteriori rule, in a way that the facies type with the largest conditional probability is allocated.

### 3.2. Cokriging of Fractions

In this approach, the fractions are first predicted and the sediment facies type is then classified according to sand content, as in the indicator approach. The kriging algorithm applied in this approach is cokriging, which can integrate multiple correlated variables. In this study, two cokriging algorithms with different input fractions are applied to compare the cases of with and without data transformation for compositional data.

First, conventional cokriging is directly applied to the original fractions of sand, silt, and clay components. The fractions are estimated via cokriging by accounting for both spatial auto- and cross-correlation information. The ordinary cokriging for estimation of the *i*th fraction values at unsampled location is written as

(4) zi∗(u)=∑α=1mλα(u)zi(uα)+∑j=2 D∑αj=1mλαj(u)zj(uα) j=1,…,D,∑α=1mλα(u)=1,∑αj=1mλαj(u)=0 j=2,…,D.

To compute the ordinary cokriging weight *λ*_*α*_*j*__ given to the* j*th component at the *α*th sample location, the direct and cross-covariance functions (equivalently, variograms) should be inferred. For two variables *z*_*i*_ and *z*_*j*_, cross-variogram, which is a quantitative measure of spatial variability between two variables, is defined as

(5) γij(h)=12N(h)×∑α=1m[zi(u)−zi(u+h)][zj(u)−zj(u+h)],

where *N*(**h**) is the number of data pairs separated by a lag distance **h**.

All direct and cross-variograms are inferred by a linear model of coregionalization (LMC), which is the way to jointly model them [5]. After generating cokriging estimates for all fractions, the same classification rule by sand content is also applied and the sediment facies types are finally obtained over the study area.

As mentioned in Section 1, the direct application of cokriging to original fractions has several drawbacks. As fractions are bounded in a simplex space, the cokriging estimates may have unrealistic negative values and do not satisfy the constant sum constraint. To solve this problem arising from the nature of compositional data, the second approach is cokriging of alr transformed fractions. By applying the alr transformation before cokriging analysis, grain size fractions in the simplex space become unbounded negative or positive values, which allows standard cokriging to be applied.

In this study, the three components of surface sediments at sample locations are transformed to their alr values as follows:

(6) $$\begin{array}{c} \text{al}{\text{r}}_{i}\left(u_{\alpha }\right)=\ln\left[\frac{z_{i}\left(u_{\alpha }\right)}{z_{D}\left(u_{\alpha }\right)}\right]\;i=1,\ldots ,D-1, \end{array}$$

where *z*_*D*_ is the arbitrarily chosen denominator of the transformation.

After alr transformation, *D* − 1 transformed variables (in our case, 2 variables) are generated and cokriging is applied to these variables. For cokriging analysis, all direct and cross-variograms are inferred from the transformed variables, not from the original fraction values. The cokriging estimates alr_i_*(**u**) for *D* − 1 transformed variables are back-transformed by taking the additive logistic transformation to ensure the constraints of compositional data as follows:

(7) $$\begin{array}{c} z_{i}^{*}\left(u\right)=\frac{\exp\left(\text{al}{\text{r}}_{i}^{*}\right)}{1+\sum_{i=1}^{D-1}\exp\left(\text{al}{\text{r}}_{i}^{*}\right)}\;i=1,\ldots ,D-1,\\ z_{D}^{*}\left(u\right)=\frac{1}{1+\sum_{i=1}^{D-1}\exp\left(\text{al}{\text{r}}_{j}^{*}\right)}. \end{array}$$

Finally, the classification rule is applied to the cokriging estimates in (7) to map the surface sediment facies types.

### 3.3. Performance Evaluation

In the case of indicator kriging of facies types, the final output is the facies type at all locations in the study area. On the other hand, cokriging of fractions, regardless of the application of alr transformation, generates two outputs: one for the fraction of each component and the other for the facies type. In this study, the prediction performance of both fractions and facies types is evaluated via leave-one-out cross-validation. One sample location is temporarily eliminated from the data sets and any kriging algorithms with the remaining samples are applied to predict either the fraction value or the facies type at the eliminated location. This procedure is repeated for all sample locations.

Two different evaluation criteria for the prediction of fractions and facies types are applied in this paper. The predictive performance of fractions, especially for the effects of alr transformation before cokriging, is quantified using the mean absolute error (MAE) for each component, defined as

(8) $$\begin{array}{c} \text{MA}{\text{E}}_{i}=\frac{1}{n}\sum_{\alpha =1}^{n}\left|z_{i}^{*}\left(u_{\alpha }\right)-z_{i}\left(u_{\alpha }\right)\right|\;i=1,2,3. \end{array}$$

The relative improvement index (RI) is also computed to quantify the effects on prediction capability of alr transformation before cokriging as

(9) $$\begin{array}{c} \text{R}{\text{I}}_{i}=100\cdot \frac{\left[\text{MA}{\text{E}}_{i}^{\text{alr}}-\text{MA}{\text{E}}_{i}^{\text{without}\;\text{alr}}\right]}{\text{MA}{\text{E}}_{i}^{\text{without}\;\text{alr}}}\;i=1,2,3, \end{array}$$

where MAE_*i*_^alr^ and MAE_*i*_^without  alr^ denote the MAE values for cokriging with and without alr transformation, respectively.

As the facies type is a categorical attribute, different evaluation criteria are applied for comparisons of facies mapping accuracy. First, a confusion matrix is first prepared, and related accuracy statistics including overall accuracy, class-wise accuracy, and kappa coefficient are calculated by comparing the true facies type and the predicted type at each sample location. The overall accuracy is the percentage of correctly classified locations in all samples, and the class-wise accuracy is a measure of the probability that a predicted sample actually represents the facies type on the true sample. The kappa coefficient is a measure of the difference between the actual agreement and the change agreement [18].

## 4. Results and Discussion

### 4.1. Exploratory Data Analysis

**Figure 2:**
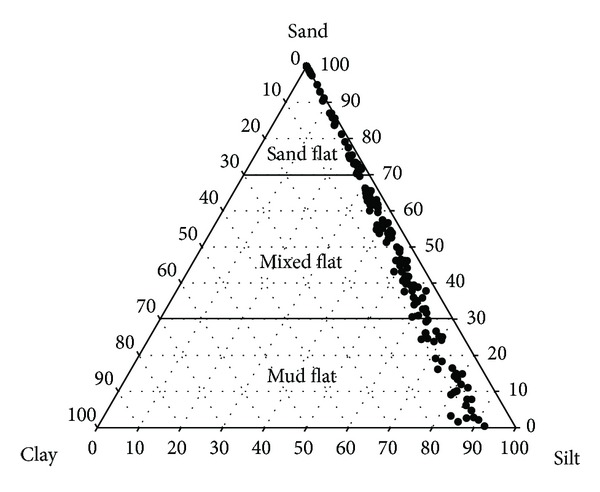
Ternary diagrams of three grain size components and sediment facies classes.

Prior to geostatistical analysis, the descriptive statistics of the grain size fractions at 174 surface sediments were computed and are summarized in Table 1. The median values for sand, silt, and clay were 60.39%, 36.43%, and 3.08%, respectively, which indicates that the majority of samples consist of coarse-grained particles. The portions for sand, mixed, and mud flats at sample locations were 39.1%, 41.4%, and 19.5%, respectively (Figure 2). As expected from the low clay content, the portion of mud flats was relatively small. Zero values in silt and clay components were observed among about 16% and 19% of all samples, respectively. The alr transformation is not defined if any observed fraction value is zero, so the zero values should be replaced by reasonable nonzero values. By following the nonparametric multiplicative strategy of Martín-Fernández et al. [19], each rounded zero in the composition was replaced by an appropriate small value and the nonzero values were then renormalized to ensure no zero values and the constant sum constraint. The small value for replacement was set to 0.1%, which was half the smallest nonzero value in the clay component. These renormalized fractions were used as inputs for the subsequent geostatistical analysis.

### 4.2. Indicator Kriging Results

Before indicator kriging, three binary indicator variables were first generated at sample locations by considering the three sediment facies types. Then, experimental indicator variograms for the three indicator variables were computed and variogram modeling was finally implemented. None of the indicator variables showed significant anisotropy, so the omnidirectional variograms were computed and modeled. The parameters of the variogram models are listed in Table 2. Especially, the indicator variogram of mixed flats was modeled with very high nugget effects and a relatively shorter range (about 1.3 km). Ordinary indicator kriging was implemented using GSLIB [17] and the order relation violation was also corrected.

**Figure 3:**
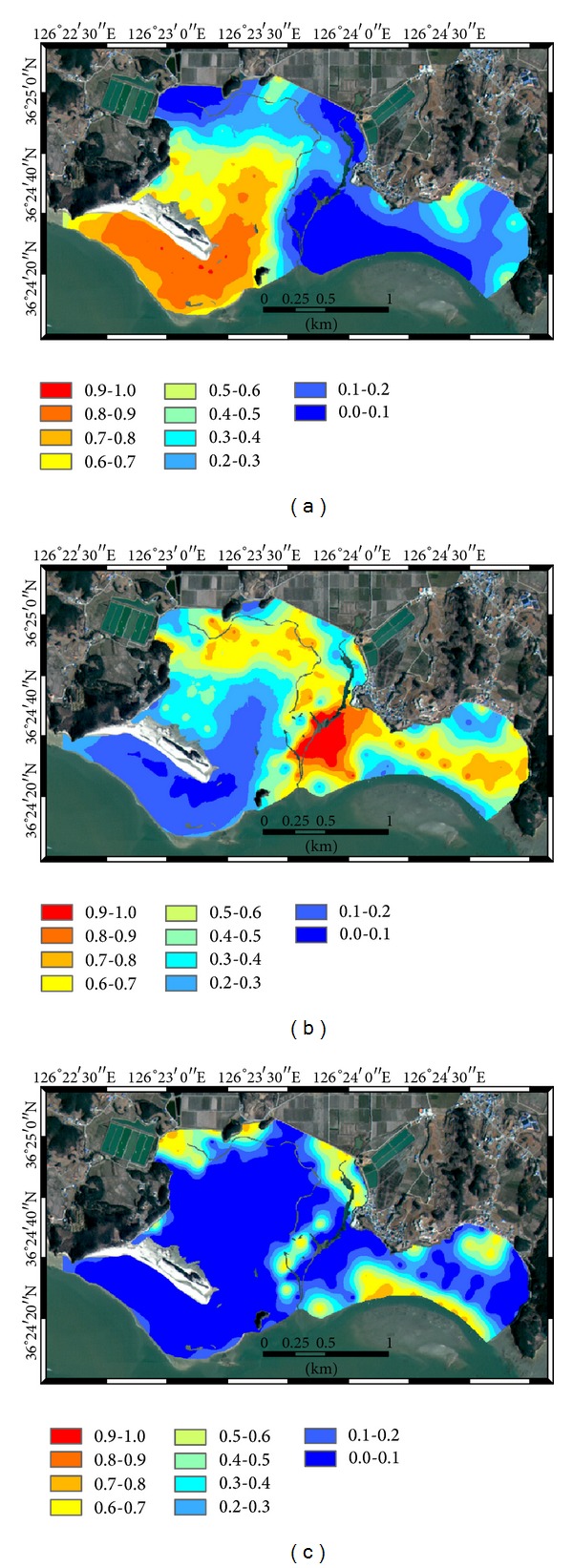
Conditional probability of each facies type prevailing over the study area by indicator kriging: (a) sand flats, (b) mixed flats, and (c) mud flats.

The conditional probabilities for the three facies types were generated on a 4 m by 4 m grid and are given in Figure 3. Sand dunes near Halmi isle and tidal channels which are not directly related to intertidal surface sediments were masked out and excluded for kriging analysis. The relatively shorter range values of the indicator variogram models led to a bull's-eye effect around the sample locations. However, the overall patterns of the conditional probability for each facies type could be observed. The conditional probability for sand flats was much higher in the southwest and northeast parts of Halmi isle. Mixed flats prevailed around the large tidal channels and showed a strong bull's-eye effect due to the relatively shorter range. Both the east seaward side and the northwest and northeast parts of the inner bay showed a relatively high probability for mud flats.

**Figure 4:**
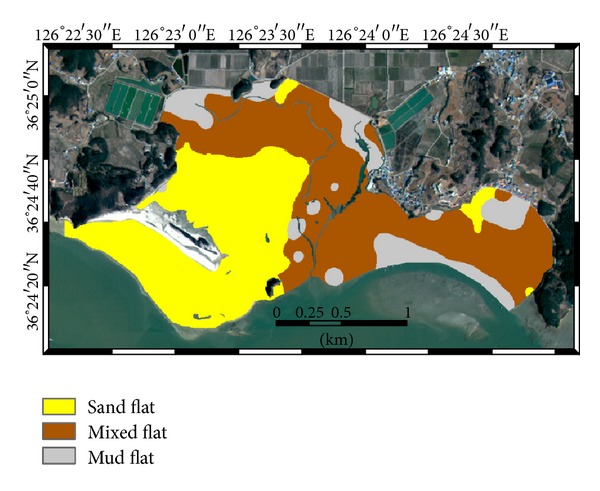
Surface sediment facies distribution generated by indicator kriging.

The final surface sediment facies types were mapped by applying the maximum a posteriori rule to the conditional probabilities and are shown in Figure 4. As expected from the conditional probabilities for the three facies types in Figure 3, sand flats are widely located at the front and back sides of Halmi isle. Mixed flats are located along the tidal channels and also at the inner bay areas. Mud flats occupy relatively small local areas in the northwest of the inner bay and the east seaward side of the study area. The mapping result also showed some spot areas such as mud flats near the tidal channels and sand flats in the southeast area. These isolated small areas are mainly located near some samples showing the intermingled patterns of conditional probabilities from mixed flats and mud flats.

### 4.3. Cokriging Results

For cokriging of the original fractions without alr transformation, three direct and two cross-variograms were modeled using the LMC. No strong anisotropy was observed, so the omnidirectional models were fitted and the parameters for the fitted models are listed in Table 3. From the variogram models and correlation coefficients, the sand component had a strong negative correlation with the silt and clay components, which indicated that, as the sand component increases, the other components decrease. The silt and clay components had a positive correlation with each other. These correlation patterns are typical characteristics of compositional data, so called spurious correlation [7, 8].

To investigate the effects of considerations on the nature of compositional data, the alr transformation was applied to raw fractions using (6). The denominator *z*_*D*_ in (6) was experimentally set to the sand component. In (6), *z*_1_ and *z*_2_ were also set to the silt and clay components, respectively. So two new alr transformed variables (i.e., alr_1_ and alr_2_) were generated and they were used as inputs for cokriging. Two direct and one cross-variograms were also modeled using the LMC. The LMC was fitted using nugget effects and an exponential model (Table 4). Although the same two basic structures were fitted for both original fractions and alr transformed variables, alr transformation led to a longer practical range of the exponential model.

**Figure 5:**
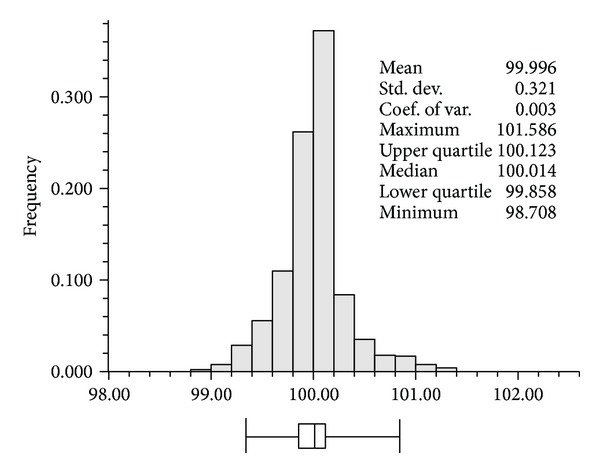
Histogram of the sums of the three fraction values at all grid locations predicted by cokriging without alr transformation. In the box plot below the histogram, outside whiskers correspond to the 95% probability intervals, inside boxes to the 50% probability intervals, and vertical lines in the box to the median value.

Cokriging was undertaken by using (4) and all variogram models. After generating cokriging estimates of the two alr transformed variables, the inverse log-ratio transformation was applied by using (7) to generate the grain size fractions in an original simplex space. Especially, the sums of the three fraction values at all grids were computed to examine if the direct application of cokriging to original fractions satisfied the constant sum constraint on compositional data. As shown in Figure 5, the sums were very close to 100%, but the constant sum constraint was not satisfied at all locations. In addition, some negative values were observed at some locations, especially in the clay component which had relatively small values. The values at any locations where negative values were estimated and/or the sum was not 100% were modified to ensure the characteristics of compositional data, in such a way that negative values or those greater than 100% were reset to 0 or 100%, respectively. Normalization was then applied to ensure the constant sum constraint. This modification procedure as postprocessing may result in some distortions or bias, thus leading to a misclassification of sediment facies types. On the contrary, the application of alr transformation before cokriging analysis perfectly satisfied the constraints of compositional data.

**Figure 6:**
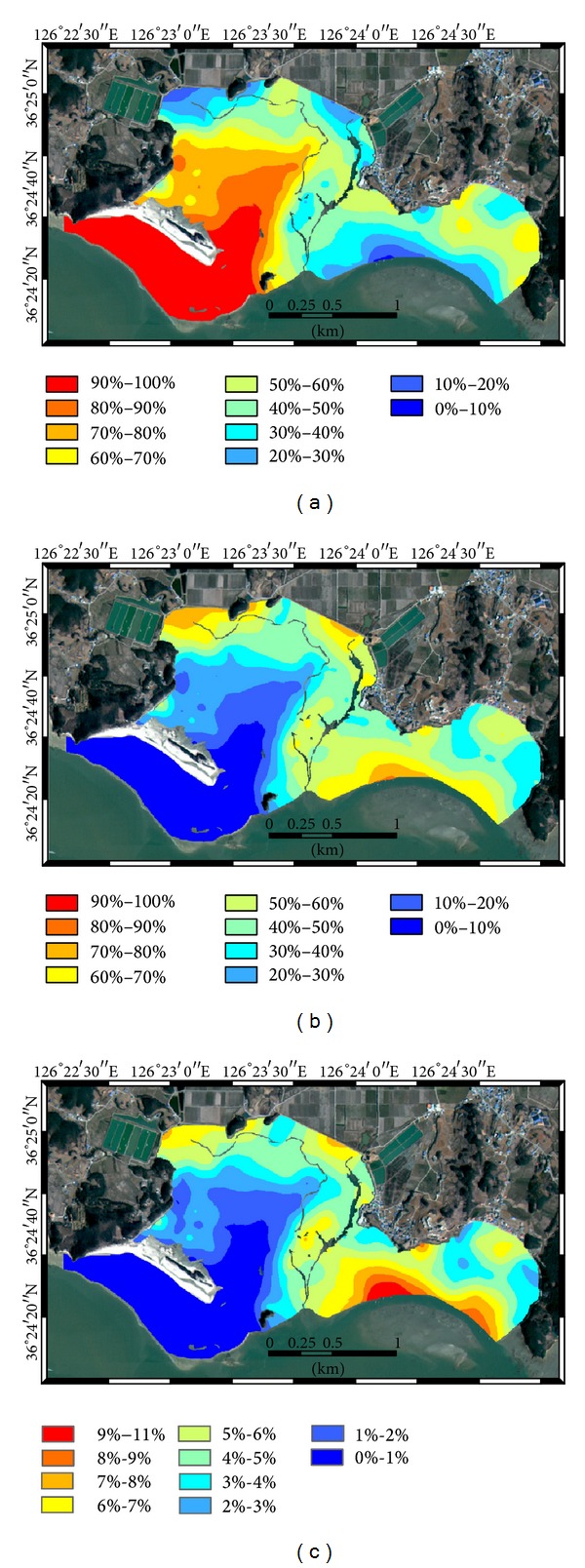
Spatial distributions of fractions generated by cokriging of original fractions: (a) sand, (b) silt, and (c) clay.

**Figure 7:**
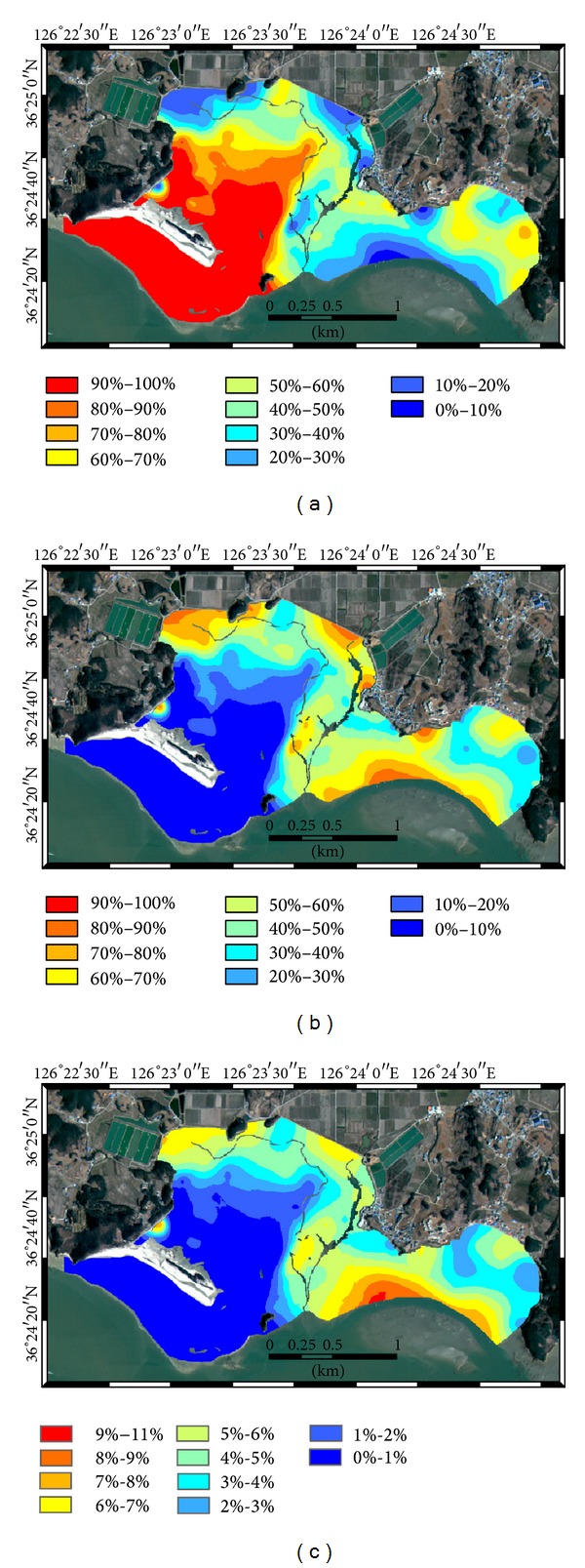
Spatial distributions of fractions generated by cokriging of alr transformed variables: (a) sand, (b) silt, and (c) clay.

The cokriging estimates for sand, silt, and clay components without and with alr transformation are shown in Figures 6 and 7, respectively. In both cokriging estimates, the overall patterns of the fractions were similar, in such a way that areas with high sand content have low content of silt and clay and vice versa. However, some locations showed slightly different values, which was attributed to the different range values of the variogram models. When comparing the sand content in Figures 6(a) and 7(a), cokriging with alr transformation led to higher and lower values of sand in the north or back side of Halmi isle and in the inner bay areas, respectively, compared with cokriging estimates without alr transformation. In the silt content estimates by cokriging with alr transformation (Figure 7(b)), low and high values were distributed more widely than in Figure 6(b). The clay content showed overall low values with high values in the east seaward side. The differences between Figures 6(c) and 7(c) for clay are distinct at the north or back side of Halmi isle, in which the low content of clay was widely distributed in Figure 7(c), compared to some areas having a relatively high content of clay in Figure 6(c).

The fractions estimated by cokriging with different inputs were directly used for sediment facies mapping. Thus these different spatial patterns of fractions led to the different mapping results and also different levels of accuracy. Leave-one-out cross-validation was carried out to quantitatively evaluate the predictive performance of the fraction estimates by the two cokriging algorithms. In the cross-validation results shown in Table 5, cokriging with alr transformation outperformed cokriging with untransformed raw fractions for all three components. The maximum relative improvement over cokriging of untransformed fractions was about 8.55% for sand. The clay component, which was the smallest content in the sediments, showed the lowest relative improvement. These cross-validation results confirmed that cokriging of alr transformed data led to reliable fractions mapping over the study area. As the predicted fractions values are directly used to map sediment facies types, the prediction accuracy of fraction values will greatly affect the facies mapping results. It should be noted that the facies mapping accuracy depends on the accuracy of all components due to the nature of the compositional data, although sediment facies mapping from predicted fractions is based only on sand content.

**Figure 8:**
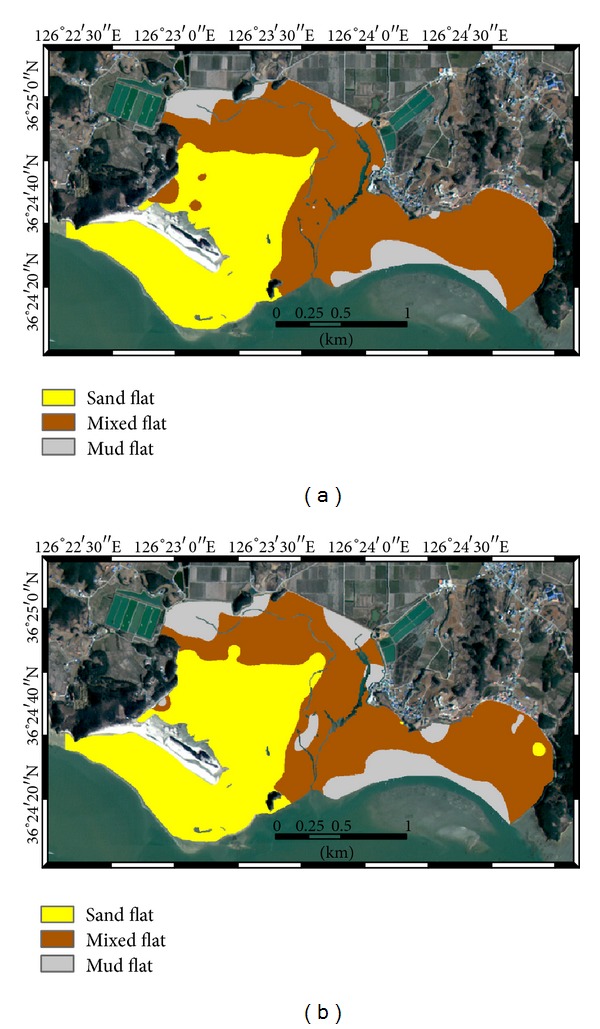
Surface sediment facies distribution generated by cokriging: (a) without and (b) with alr transformation.

The sediment facies distributions based on the two different cokriging algorithms were generated by using the same classification rule (i.e., sand content). The mapping results are presented in Figure 8. As expected from the wide distribution of sand and silt in Figure 7, cokriging with alr transformation generated the relatively wide distributions of both sand flats around Halmi isle and mud flats in the inner bay. When compared with the facies distribution generated by indicator kriging shown in Figure 4, some spots classified into mud flats near the tidal channels in Figure 4 disappeared in both Figures 6 and 7. As kriging is an exact interpolator, it honors data values at sample locations [5]. Thus, the facies types at sample locations are reproduced in all maps by any kriging algorithms. However, the use of different mapping procedures led to the different mapping results near the sample locations. In the facies mapping result based on indicator kriging, spatial correlation structures of facies types are directly used in indicator kriging, so any locations near samples tend to have the same facies type, depending on the correlation strength. Meanwhile, the facies mapping scheme based on cokriging of fractions first generated fraction estimates over the study area and the mapping rule was then applied as a final stage. Thus, spatial correlation structures of the fraction values are only considered for the mapping of fractions and relatively few isolated locations were mapped.

The following common or overall patterns of sediment facies distributions were observed over the study area in Figures 4 and 8. Sand flats are mainly located near Halmi isle; especially, the front or southern areas of Halmi isle are open to the outer sea, so sediments in these areas have been deposited under the depositional environments with strong sediment supply. Mixed flats, which are located along the large tidal channels at the east of the study area, are affected by both tidal currents and ocean waves. The mud flats observed at the inner bay were developed by the reversing tidal currents or the decrease of depositional energy due to the topographic effects. The large sand bar located in the southeastern part of the study area has blocked the wave energy, allowing fine-grained sediments to be continuously deposited, and mud flats developed at the east seaward side of the study area.

### 4.4. Mapping Accuracy Assessment

Like the comparison of the predictive performance of cokriging with different input data, leave-one-out cross-validation was also implemented to quantitatively assess facies mapping accuracy. For indicator kriging, conditional probabilities at a temporarily eliminated sample location were estimated by the remaining sample data. Then the MAP rule was also applied to determine the facies type at the eliminated location. For two cokriging algorithms, fraction values predicted at all sample locations have already been obtained from leave-one-out cross-validation. So the sediment facies types at all sample locations were determined based on the cross-validated sand content. True facies types at sample locations, which are required to construct a confusion matrix, were also determined by applying the same classification rule.

Several statistics were computed from the confusion matrix as quantitative measures of accuracy assessment and are listed in Table 6. Facies mapping based on cokriging of alr transformed variables showed the best mapping accuracy in terms of both overall accuracy and kappa coefficient, which achieved 7.5%p and 9.8%p increases in overall accuracy compared with that from cokriging of untransformed fractions and that from the indicator kriging approach, respectively. The indicator approach afforded the worst mapping accuracy. The class-wise accuracy was also the best in the mapping results from cokriging with alr transformation, except for the mud flats in which cokriging without data transformation showed slightly better accuracy. These evaluation results confirmed the effectiveness of and necessity for the prediction of fraction values with data transformation as a first stage and the subsequent mapping for sediment facies mapping. Especially, the characteristics of compositional data should be considered for spatial prediction of fraction values and the prediction results affected the final facies mapping results.

The worst mapping accuracy by the indicator kriging approach can be explained as follows. The indicator kriging approach requires the predefinition of facies types at sample locations. As shown in the ternary diagram of Figure 2, each facies type occupies a certain zone in the ternary diagram, so the sediment classified as the same facies type can have very different sand contents. For example, two sediment samples having a sand content of 31% and 69%, respectively, are classified as the same mixed flats type. However, the characteristics of the former and latter sediments are similar to those of mud flats and sand flats, respectively. Thus using the predefined class cannot account for the variation of fraction values within each facies type. This variation within the facies type may result in the confusion between nearby facies types within the ternary diagram.

Another limitation of the indicator kriging approach is its inability to represent all possible facies types not observed at sample locations. In this study, the three facies types were experimentally predefined and mapped according to sand content. Other classification or mapping rules can be applied, for example, Shepard's rule [3]. These classification rules are based on the relative proportions among sand, silt, and clay. For example, according to Shepard's classification, if the clay content is less than 20%, the sand content is less than 75% and is greater than the silt content, and the silt content is greater than the clay content, the sediment is classified as silty sand. When considering this classification rule, the small number of sediment samples which is a typical case of data collection in tidal flats may result in several practical issues. If the above classification rule is applied to very few samples, some sediment types may not be contained among the predefined types from the sample data and may, therefore, not exist in the final mapping result, although they should be included. For the application of indicator kriging, it may be difficult or even impossible to capture spatial patterns and model variograms of a certain sediment type in which very few samples are classified. The mapping method based on the prediction of fractions before applying any classification rules can overcome these limitations of the indicator kriging approach.

## 5. Conclusions

Three different kriging algorithms have been compared and evaluated for intertidal surface sediment facies types. A case study using grain size samples collected in the Baramarae tidal flat, Korea, indicated that the prediction of fractions values in advance and the subsequent classification improved mapping accuracy, compared to that from the direct prediction of predefined facies types via indicator kriging. Thus, a prerequisite is to generate reliable predictions of grain size fractions for facies mapping. When comparing two cokriging algorithms with different variables, spatial prediction based on cokriging of log-ratio transformed variables could satisfy all constraints for compositional data and produce not only better accuracy for the prediction of fractions, but also the best mapping accuracy, when compared with spatial prediction based on cokriging of raw fraction values. Thus, the prediction of fraction values, which can account for the characteristics of grain size fractions as compositional data, should be applied before cokriging analysis to generate reliable facies mapping results.

From a methodological point of view, the major finding in this paper can be further extended to improve facies mapping accuracy. When sparse sediment samples are available, as in the case in tidal flats, more densely sampled or exhaustively sampled auxiliary data, which are related to the characteristics of grain size data, can improve the prediction quality. Remote sensing data [1, 2] or terrain data [20, 21] can be effectively used to improve the prediction of grain size or sediment facies. When integrating these auxiliary data with grain size data, however, the characteristics of compositional data should still be considered. Although previous studies [2, 20, 21] reported the ability of terrain data to complement the sparse grain size data, the proper processing of grain size data as compositional data was not considered. The integration of alr transformed fractions with high-resolution remote sensing data will be considered in future work.

## Figures and Tables

**Table 1:** Summary statistics of grain size fractions in all samples.

Statistic	Component		
	Sand	Silt	Clay
Mean	59.14	37.23	3.63
Standard deviation	30.42	27.48	3.17
Minimum	0.35	0.00	0.00
25% quantile	36.41	12.54	0.83
Median	60.39	36.43	3.08
75% quantile	86.86	58.44	5.78
Maximum	100.00	92.35	13.93
Skewness	−0.16	0.14	0.68
Portion of zero values (%)	0.00	16.09	18.97

**Table 2:** Parameters for indicator variogram models.

Facies type	Model	Nugget	Partial sill	Range (m)
Sand flats	Exponential	0.103	0.223	3015
Mixed flats	Spherical	0.186	0.065	1029
Mud flats	Spherical	0.099	0.295	7575

**Table 3:** Parameters for the fitted linear model of coregionalization for original three components.

Model	Variance-covariance matrix				Range (m)
Nugget		Sand	Silt	Clay	—
	Sand	210.005			
	Silt	−175.013	175.022		
	Clay	−17.001	16.051	2.201	

Exponential		Sand	Silt	Clay	1386
	Sand	697.460			
	Silt	−630.652	570.252		
	Clay	−68.990	62.292	7.684	

**Table 4:** Parameters for the fitted linear model of coregionalization for alr.

Model	Variance-covariance matrix			Range (m)
Nugget		alr_1_	alr_2_	—
	alr_1_	1.332		
	alr_2_	0.911	1.116	

Exponential		alr_1_	alr_2_	2908
	alr_1_	15.381		
	alr_2_	13.069	11.865	

**Table 5:** Predictive performance of fraction estimates by different cokriging algorithms.

Component	MAE		RI (%)
	Cokriging without alr transformation	Cokriging with alr transformation	
Sand	15.71	14.36	8.55
Silt	14.17	13.13	7.34
Clay	1.71	1.62	5.27

**Table 6:** Mapping accuracy statistics by different mapping algorithms.

Statistics	Indicator kriging	Cokriging without alr transformation	Cokriging with alr transformation
Overall accuracy (%)	63.79	66.09	73.56
Kappa coefficient	0.42	0.44	0.58
Class-wise accuracy (%)			
Sand flats	80.65	83.33	83.61
Mixed flats	55.43	56.48	65.85
Mud flats	50.00	75.00	74.19
